# Supplementary material for: Simulated-to-real benchmarking of acquisition methods in untargeted metabolomics
Source: Front Mol Biosci. 2023 Mar 7;10:1130781. doi: 10.3389/fmolb.2023.1130781 (PMC10027714; doi:10.3389/fmolb.2023.1130781)
Supplement: Supplementary file 1 [file DataSheet1.PDF]

Supplementary Material

SUPPLEMENTARY SECTION S1: BEER SAMPLES

The following a list of beer samples used:

Index	Name	Type
1	Raspberry Sour by Vault City	Sour
2	Cacao & Hazelnut Broken Dream Twisted Breakfast Stout by Siren	Stout
3	Tennents	Lager
4	Life and Death by Vocation	IPA
5	Silence is... Citra by Overtone	Pale Ale
6	Punk AF by Brewdog	Alcohol-Free IPA

Table S1. The list of beers used in the experiment.

SUPPLEMENTARY SECTION S2: MS-DIAL PARAMETERS FOR SIMULATED DATA

The following are MS-DIAL parameters used to process the DIA (SWATH, AIF) mzML files generated in simulation:

```
#Data type
MS1 data type: Centroid
MS2 data type: Centroid
Ion mode: Positive
DIA file: {config.txt}

#Data collection parameters
Retention time begin: 0
Retention time end: 10
Mass range begin: 0
Mass range end: 1100

#Centroid parameters
MS1 tolerance for centroid: 0.01
MS2 tolerance for centroid: 0.05

#Peak detection parameters
Smoothing method: LinearWeightedMovingAverage
Smoothing level: 3
Minimum peak width: 5
Minimum peak height: 1000
Mass slice width: 0.05

#Deconvolution parameters
Sigma window value: 0.5
Amplitude cut off: 10

#Adduct list
Adduct list: [M+H]+

#MSP file and MS/MS identification setting
MSP file: {msp_file}
Retention time tolerance for identification: 100
Accurate ms1 tolerance for identification: 0.025
Accurate ms2 tolerance for identification: 0.25
Identification score cut off: 0

#Text file and post identification (retention time and accurate mass based) s[illegible]
#Text file:
Retention time tolerance for post identification: 0.1
Accurate ms1 tolerance for post identification: 0.01
```

```
Post identification score cut off: 85

#Alignment parameters setting
Retention time tolerance for alignment: 0.05
MS1 tolerance for alignment: 0.0015
Retention time factor for alignment: 0.5
MS1 factor for alignment: 0.5
Peak count filter: 0
QC at least filter: True

#CorrDec setting
CorrDec excute: False
```

For AIF, {config.txt} is the following:

```
ID MS Type Start m/z End m/z Name CE DecTarget(1:Yes, 0:No)
0 SCAN 0 1100 0eV 0 0
1 ALL 0 1100 30eV 30 1
```

For SWATH, {config.txt} is the following:

```
Experiment MS Type Min m/z Max m/z
0 SCAN 0 1100
1 SWATH 0 100
2 SWATH 100 200
3 SWATH 200 300
4 SWATH 300 400
5 SWATH 400 500
6 SWATH 500 600
7 SWATH 600 700
8 SWATH 700 800
9 SWATH 800 900
10 SWATH 900 1000
11 SWATH 1000 1100
```

SUPPLEMENTARY SECTION S3: MS-DIAL PARAMETERS FOR BEER SAMPLES

The following are MS-DIAL parameters used to process the DIA (SWATH, AIF) mzML files generated from actual beer samples produced on the Thermo instrument:

```
#Data type
MS1 data type: Centroid
MS2 data type: Centroid
Ion mode: Positive
DIA file: {config.txt}

#Data collection parameters
Retention time begin: 3
Retention time end: 24
Mass range begin: 70
Mass range end: 1100

#Centroid parameters
MS1 tolerance for centroid: 0.01
MS2 tolerance for centroid: 0.05

#Peak detection parameters
Smoothing method: LinearWeightedMovingAverage
Smoothing level: 3
Minimum peak width: 5
Minimum peak height: 25000
Mass slice width: 0.05

#Deconvolution parameters
Sigma window value: 0.5
Amplitude cut off: 10

#Adduct list
Adduct list: [M+H]+

#MSP file and MS/MS identification setting
#MSP file: {msp_file}
Retention time tolerance for identification: 100
Accurate ms1 tolerance for identification: 0.025
Accurate ms2 tolerance for identification: 0.25
Identification score cut off: 0

#Text file and post identification (retention time and accurate mass based) s
#Text file: D:\Msdial-ConsoleApp-Demo files\Msdial-ConsoleApp-Demo files for
Retention time tolerance for post identification: 0.1
Accurate ms1 tolerance for post identification: 0.01
```

```
Post identification score cut off: 85

#Alignment parameters setting
Retention time tolerance for alignment: 0.16666666666
MS1 tolerance for alignment: 0.025
Retention time factor for alignment: 0.5
MS1 factor for alignment: 0.5
Peak count filter: 0
QC at least filter: True

#CorrDec setting
CorrDec excute: False
```

For AIF, {config.txt} is the following:

```
ID MS Type Start m/z End m/z Name CE DecTarget(1:Yes, 0:No)
0 SCAN 70.0 1100.0 0eV 0 0
1 ALL 70.0 1100.0 25eV 25 1
```

For SWATH, {config.txt} is the following:

```
Experiment MS Type Min m/z Max m/z
0 SCAN 70.0 1100.0
1 SWATH 70 170
2 SWATH 170 270
3 SWATH 270 370
4 SWATH 370 470
5 SWATH 470 570
6 SWATH 570 670
7 SWATH 670 770
8 SWATH 770 870
9 SWATH 870 970
10 SWATH 970 1070
```

SUPPLEMENTARY SECTION S4: INTENSITY NON-OVERLAP ACQUISITION METHOD

Intensity Non-overlap is an advanced iterative DDA-based method that incorporates several new concepts to achieve more targeted fragmentations and prevent more redundant ones across samples or injections. This incorporates several key ideas to greatly increase the fragmentation coverage of unique molecular features across multiple injections. First is the method incorporates the concept of tracking Region of Interest (RoI, groups of related precursor ions) in real-time and prioritising RoIs for fragmentation rather than individual ions. Then across repeated injections, the same RoIs if seen again are downweighted by their overlapping area. Finally its score component also prioritises acquiring the same RoI again if it can be fragmented at a higher apex than before as compared to the previous injection. Intensity Non-overlap is builds upon ‘TopNEXt’ [1], a real-time scan prioritisation framework and an extension of the Virtual Metabolomics Mass Spectrometer which implements several improved multi-sample fragmentation strategies within a modular and cohesive base. For more details, please refer to [1].

SUPPLEMENTARY SECTION S5: MASS DISTRIBUTION OF SAMPLED HMDB CHEMICALS

Figure S1 shows a kernel density plot of the monoisotopic masses of the 5000 chemicals sampled from HMDB (restricted between 100-1000 Da) for one replicate in the simulated experiment. The other 4 replicates in the experiment broadly follow the same pattern.

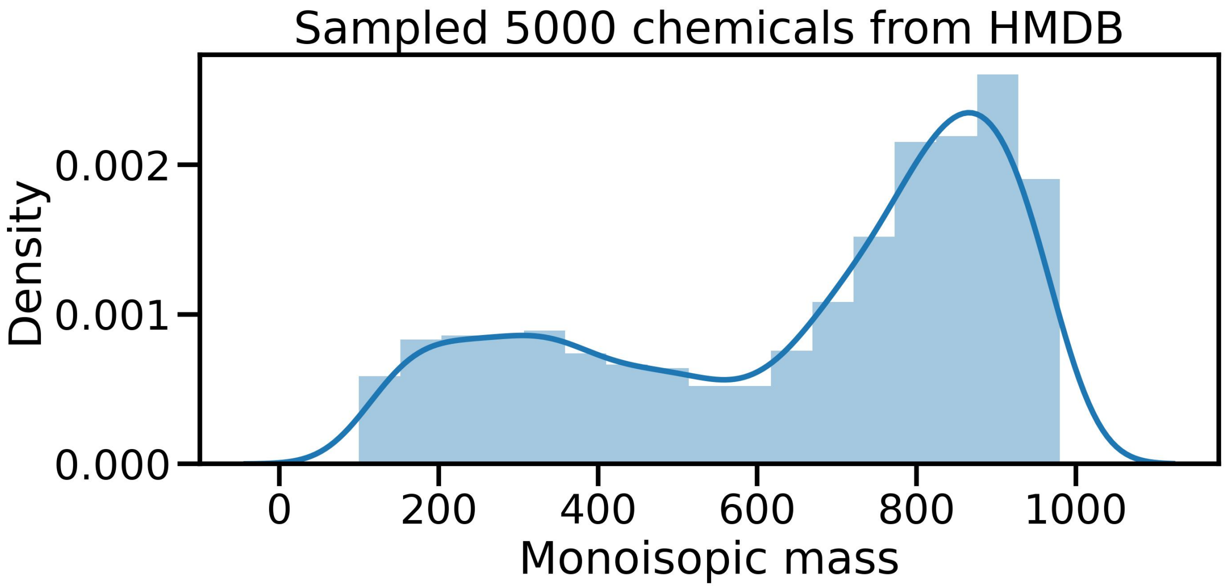

Figure S1: Mass distribution of 5000 chemicals sampled from HMDB

SUPPLEMENTARY SECTION S6: DIFFERENT THRESHOLDS FOR MATCHING

The following are results from using different cosine similarity thresholds when matching the simulated chemicals with the observed DDA and DIA spectra. Figure S2 uses a bin width of 0.005 Da, while Figure S3 uses a bin width of 0.50 Da.

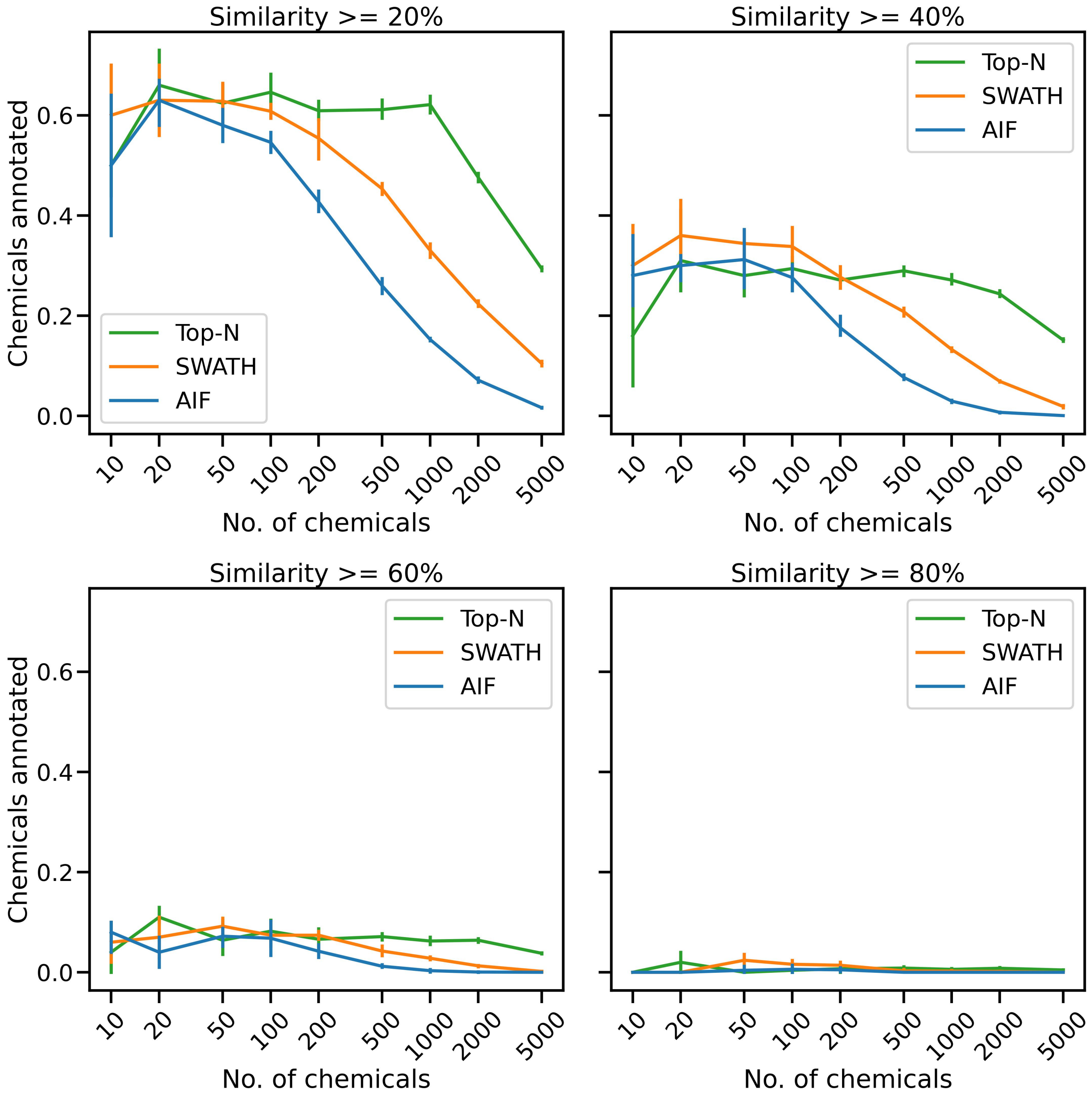

Figure S2: The mean proportion of unique chemical annotations at varying numbers of chemicals and similarity thresholds across 5 replicates. The error bar shows the 95% confidence interval. MS2 matching was done using a bin width of 0.005 Da and a minimum of 3 matching peaks.

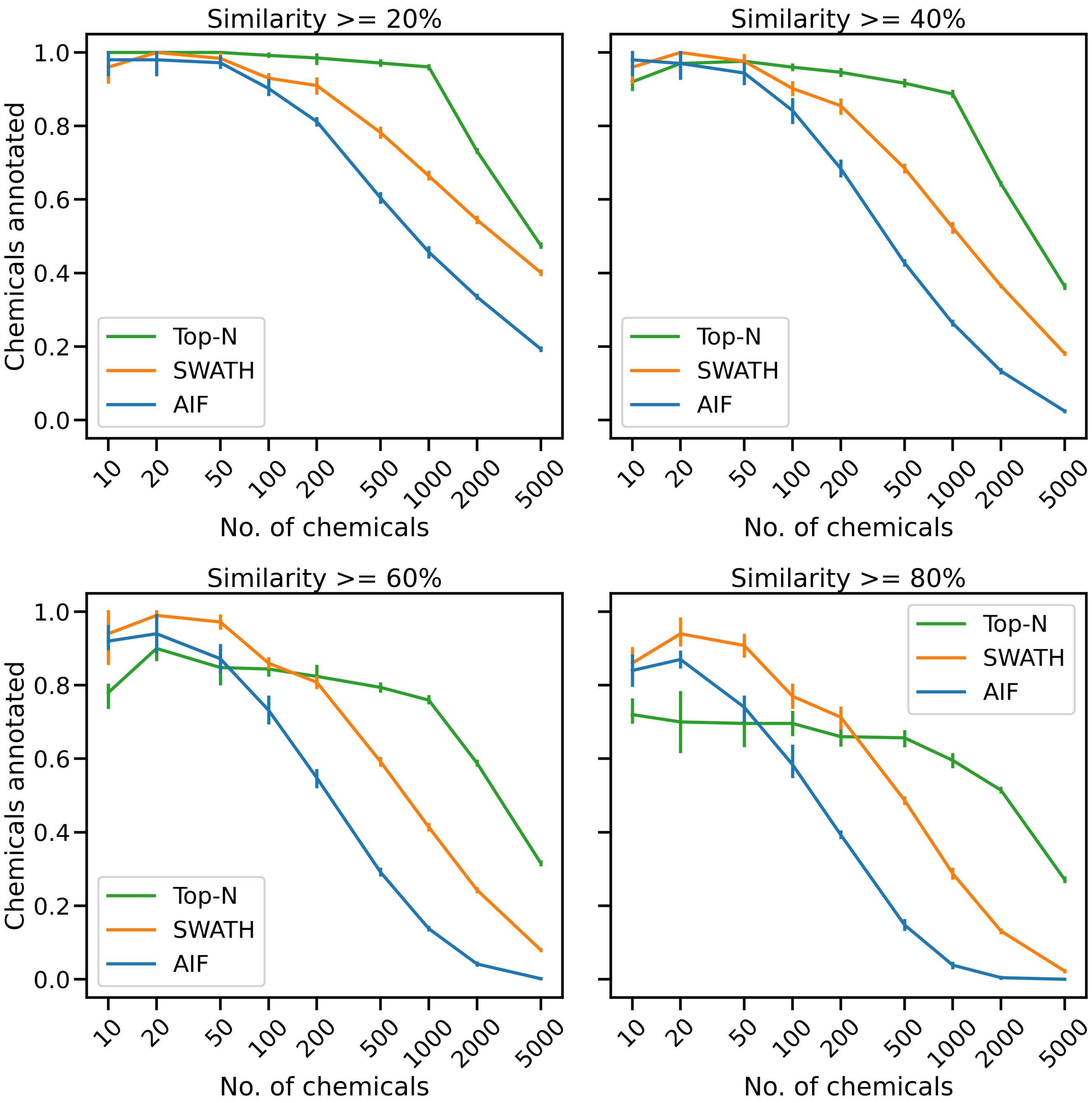

Figure S3: The mean proportion of unique chemical annotations at varying numbers of chemicals and similarity thresholds across 5 replicates. The error bar shows the 95% confidence interval. MS2 matching was done using a bin width of 0.50 Da and a minimum of 3 matching peaks.

SUPPLEMENTARY SECTION S7: DIFFERENT WINDOWS FOR SWATH

Figure S4 shows the results from simulating different window sizes for SWATH. Three different window sizes of 100 m/z, 50 m/z and 25 m/z were used in the simulations for two similiarity thresholds of $\geq 60\%$ and $\geq 80\%$ used for the evaluation of matching quality. It can be observed that making the window size smaller produces a slight annotation improvement when the number of chemicals are 2000 and 5000. We have also included Top-N (DDA) as the baseline.

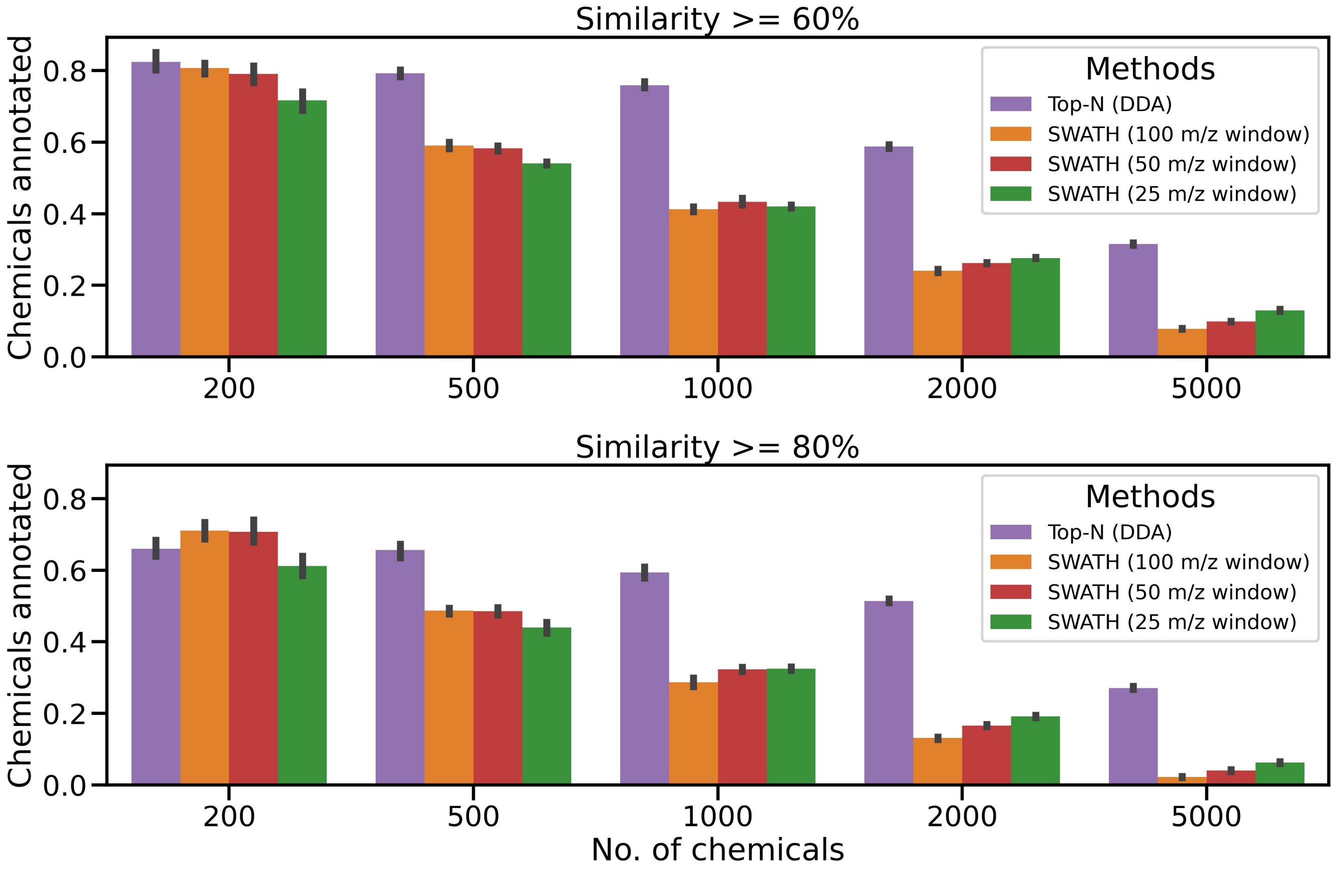

Figure S4: The mean proportion of unique chemical annotations for 200, 500, 1000, 2000 and 5000 chemicals across 5 replicates for different SWATH window sizes at similarity thresholds of $\geq 60\%$ and $\geq 80\%$.

REFERENCES

[1]R. McBride, J. Wandy, S. Weidt, S. Rogers, V. Davies, R. Daly, and K. Bryson. TopNEXt: Automatic DDA exclusion framework for multi-sample mass spectrometry experiments. *bioRxiv*, 2023.
